# Beneficial effects of ROCEN (Topical Nano-arthrocen) on atopic dermatitis in mice

**DOI:** 10.1186/s12906-021-03393-0

**Published:** 2021-09-06

**Authors:** Ramin Goudarzi, Maryam Eskandarynasab, Ahad Muhammadnejad, Ahmad Reza Dehpour, Alireza Partoazar

**Affiliations:** 1 Division of Research and Development, Pharmin USA, LLC, San Jose, California USA; 2 Department of Pharmacology, School of Medicine, Tehran University of Medical Sciences, Tehran, Iran; 3 Cancer Biology Research Center, Cancer Institute of Iran, Tehran University of Medical Sciences, Tehran, Iran; 4 Experimental Medicine Research Center, Tehran University of Medical Sciences, Tehran, Iran

**Keywords:** Atopic dermatitis, Avocado and soybean, Oxazolone, Nanocen, IL-8, TNF-α

## Abstract

**Objective:**

Atopic dermatitis (AD) is a chronic inflammatory skin disease mainly caused by immune stimuli. The current study was conducted to investigate the effects of ROCEN and to compare it with betamethasone (Beta) on mice subjected to AD.

**Methods:**

First, the safety of topical ROCEN was tested to determine possible sensitization induction in vivo. Then, the mice were subjected to oxazolone (Oxa) to induce chronic AD. Consequently, they underwent treatment with ROCEN and Beta. Scratching and wiping behaviors related to dermatitis were evaluated in treated animals for 35 days. The histopathology and immunohistochemistry (IHC) analysis of interleukin-8 (IL-8) and tumor necrosis factor-α (TNF-α) cytokines were performed on the dorsal skin of the treated mice.

**Results:**

Topical administration of ROCEN and Beta to the dorsum of sensitized mice for 5 weeks significantly alleviated scratching and wiping symptoms and reduced erythema, scaling, and edema in the skin of the mice with AD. Moreover, histological indices showed that ROCEN effectively reduced leucocyte infiltration and improved skin healing parameters in treated AD mice. Application of ROCEN or Beta reduced IHC markers including IL-8 and TNF-α significantly.

**Conclusion:**

ROCEN alleviated the AD symptoms similar to betamethasone in an experimental animal model.

## Introduction

Atopic dermatitis (AD) is a highly prevalent, chronic, inflammatory skin disease [1] characterized by severe itching, a mild to severe rash, edema, hemorrhage, and erosion of the skin surface [2]. It is associated with a variety of immunological mechanisms and environmental and even neuropsychological factors may have a role in the development of this disease [3–6].

One of the characteristic features of AD is the involvement of various inflammatory cells including lymphocytes, eosinophils, and neutrophils with a predominant Th_2_ cell response [7, 8]. An allergen exposure may stimulate keratinocytes and release cytokines like TNF-α, resulting in the excitation of proinflammatory cytokines production including IL-1b, IL-6, and IL-8 from other keratinocytes and dermal cells [8]. This process can be directed to the allergic phase via recruitment and activation of leukocytes and dendritic cells and presenting the allergen to specific T-cells. It has been shown that IL-8 cytokine production by Th_2_ cells activated by allergic contact response can contribute to activation and regulation of various immune cells in inflamed tissues [8, 9].

The skin barrier function is disrupted in AD, which is under the influence of lipids located within the stratum corneum (SC) in the epidermis. It is believed that pro-inflammatory cytokines such as TNF-α decrease the level of long-chain free fatty acids and ceramides, which consequently affects the lipid organization distribution and results in skin injury [10]. Barrier dysfunctionality in AD accelerates the penetration of allergens into the skin layers, resulting in immune response stimulation [11].

Chronic AD, eczema, is mostly treated with corticosteroids like topical Beta [12] while its long-term use can cause serious side effects including hyperglycemia and dermal and epidermal injuries [13]. Therefore, new formulations and drugs are always of interest to improve the quality of life of AD patients.

Arthrocen (ASU), an oil extract of avocado and soybean, has been shown to downregulate inflammatory mediators such as TNFα, IL8, and PE2 and increase the expression of transforming growth factor-beta (TGF-β) and production of collagen and aggrecan in the connective tissue [14, 15]. Evidence [15, 16] suggests that topical ASU, ROCEN, may regulate inflammatory cells and cytokines and improve tissue injury due to eczema. However, because of the very low water solubility of ASU, the use of pharmaceutical carriers such as liposomes is recommended for effective skin delivery of lipophilic molecules of the drug [17–19]. Liposome carriers not only are versatile tools in drug delivery, but also have anti-oxidative, anti-inflammatory, and wound repair properties [20–22].

We recently used a topical formulation of liposomal ASU to enhance the drug efficacy in terms of wound healing, inflammation reduction, and pain alleviation in animal models [16, 19]. In a study by Goudarzi et al., ROCEN formulation potentially caused TGF-β1 production in burnt tissues of rats, resulting in wound healing acceleration and thermal pain alleviation with a long-lasting effect [16]. Hence, this study was conducted to investigate the effects of the topical.

## Materials and methods

### Drug preparation

Liposomal formulation of ASU was prepared according to our recent study [16]. Briefly, ASU (2% w/v) with the appropriate lipids were dissolved in ethanol. Then phosphate buffer saline was added to the mixture at 40 °C and under stirring condition (700 rpm). Eventually, the suspension was homogenized with mild power for 5 min to obtain ROCEN formulation. Oxazolone (Oxa) was obtained from Sigma-Aldrich (USA), ASU manufactured by Pharmin USA, LLC (USA). Beta %0.1 was prepared from Sobhan Daru Pharmaceutical Company (Iran).

### Animal

The female BALB/c mice (mean weight 30 ± 2 g) were procured from Tehran University of Medical Sciences (Tehran, Iran). The animals were placed under controlled environmental conditions at 23 ± 1 °C with 50% humidity and 12 h dark/light cycles. The animals were subjected to acclimatization for at least 1 week before the experiment.

### Safety evaluation of ROCEN

**Fig. 1:**
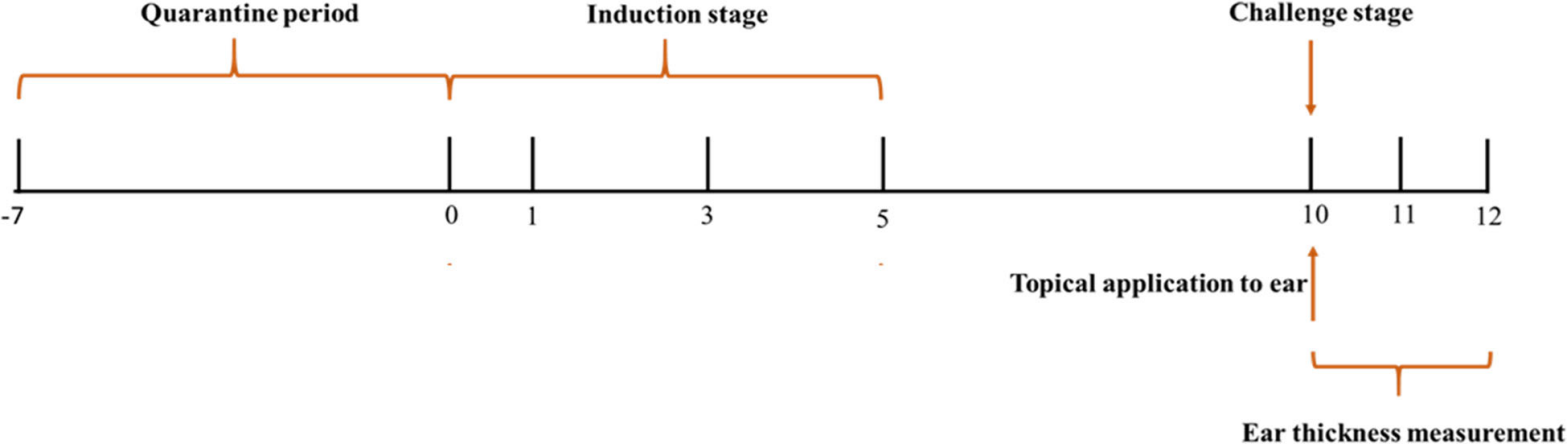
The line chart of the MEST protocol for allergenicity assessment of the drug

In our study, a primary delayed-type contact hypersensitivity test, mouse ear swelling test (MEST), was done for evaluation of possible allergic contact dermatitis due to ROCEN application. Briefly, mice were divided randomly into 3 groups consisted of 10 mice. The groups included control, ROCEN, formaldehyde (positive control). The test substance was judged to be a non-irritant agent before the hypersensitivity test and then the experiment was followed by a standard protocol that has been depicted briefly in Fig. 1. A positive sensitization response was concerned to happen if the one or more ear test of mice was at least 20% thicker than the control ear [23].

### Experimental design

**Fig. 2:**
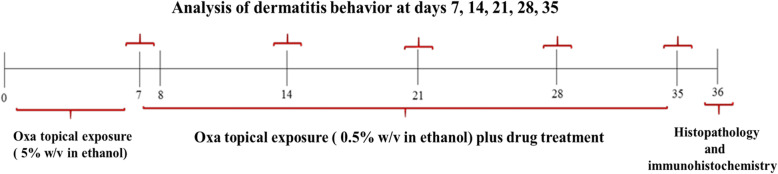
Summary of experimental dermatitis protocol investigated on AD mice

The mice were randomly divided into the four groups with 6 animals in each experiment and defined as follows: Sham group: vehicel, Oxa group: Oxa 60 μg/ day + Water 100 μl/ day, ROCEN group: Oxa 60 μg/ day + ROCEN 100 μl/ day, Beta group: Oxa 60 μg/ day + Beta 1%, 100 μl/ day. In all experimental days (0–35), skin severity and behavior of animals due to skin dermatitis were evaluated according to the below sections. One day after the last dose of the drug (36th day), animals were euthanized with CO_2_ and then their dorsal skins were separated for histopathology and IHC analysis. A brief description of the experimental is individual in Fig. 2.

### Induction of dermatitis and treatment

In this study, AD was induced in mice according to the Goindi et al. method [24]. Firstly, female BALB/c mice were anesthetized with ketamine (50 mg/kg) and xylazine (10 mg/kg) and the hair on the upper back of each animal was shaved 1 day before the experiments. Topical application of 10 μl Oxa (5% w/v in ethanol) on the shaved dorsal skin was continued daily for 1 week. Then, the same experiments were followed daily by gently rubbing of 60 μl Oxa (0.5% w/v in ethanol) with a frequency of 30× per min on the dorsal skin for the 4 weeks. 90 min after Oxa application, drug was administered topically by rubbing with a frequency of 50× per min on the animals’ dorsum in treated groups. In the sham group, only 60 μl ethanol (without Oxa) was applied to the dorsum of mice.

### Evaluation of skin severity

The severity of AD on the dorsal skin was scored according to the mean of total symptoms including erythema, scarring/dryness, edema, and excoriation/erosion once a week. The symptom of erythema was scored as 0 (none), 1 (very slight (light pink)), 2 (well-defined (dark pink)), 3 (moderate to severe (light red)), 4 (severe (dark red)). The other relative symptoms were scored by numbers of 0 (none), 1 (mild), 2 (moderate), 3 (severe) [25].

### Measurement of scratching/wiping behavior

Mice were placed individually in the acrylic cages with the four cells in dimensions of 15 cm × 10 cm × 10 cm. Small openings were created in each cell to allow air circulation. The camera recorder was embedded in the front of the cages to record animals’ behavior. The adaptation of mice to the experimental conditions was done by handling, restraining, and placing them in containers several times before the experiments began on each experimental day. On the 7th, 14th, 28th, and 35th days of the experiment, the animals’ behavior at 90th min after drug application was recorded by the camera for 20 min. Scratching, grooming, and licking behaviors were assessed according to the frequency of each behavior during 20 min experiment counted from video records [26].

### Histopathology and immunohistochemistry

Tissue samples were taken from dermatitis areas of mice dorsum on the 36th day of the experiment. Skin samples were fixed in 10% (v/v) formalin buffer with PBS and then dehydrated and embedded in paraffin. The section of samples was prepared with 5 μm thickness and stained with hematoxylin and eosin for histopathology analyses. An expert pathologist assessed as blindness the sections for epithelial thickness, collagen deposition, angiogenesis, leukocyte infiltration, mononuclear cells (MCs), lymphocytes (Lymphs)/Plasma cells (PCs) using a light photomicroscope.

The tissue slides were also subjected to the immunohistochemical assay of TNF-α (orb39299, biorbyt, Cambridge (United Kingdom)), and IL-8 (orb39299, biorbyt, Cambridge (United Kingdom)) using commercially available antibodies according to the manufacturers’ instructions and then analyzed by the pathologist [27]. All slides were analyzed in a blinded fashion by an expert pathologist and cytokines level of brain samples were scored as follows: negative (0–1), weak (2–3), moderate (4–6), and severe (7–8). The presence of brown cells in the stained sections indicated the expression of the relative cytokine markers [28].

### Statistical analysis

Statistical analysis of data was performed by one-way analysis of variance (ANOVA) to compare the significant differences among each group. *P* < 0.05 was considered as statistically significant.

## Results

### Safety of ROCEN

**Fig. 3:**
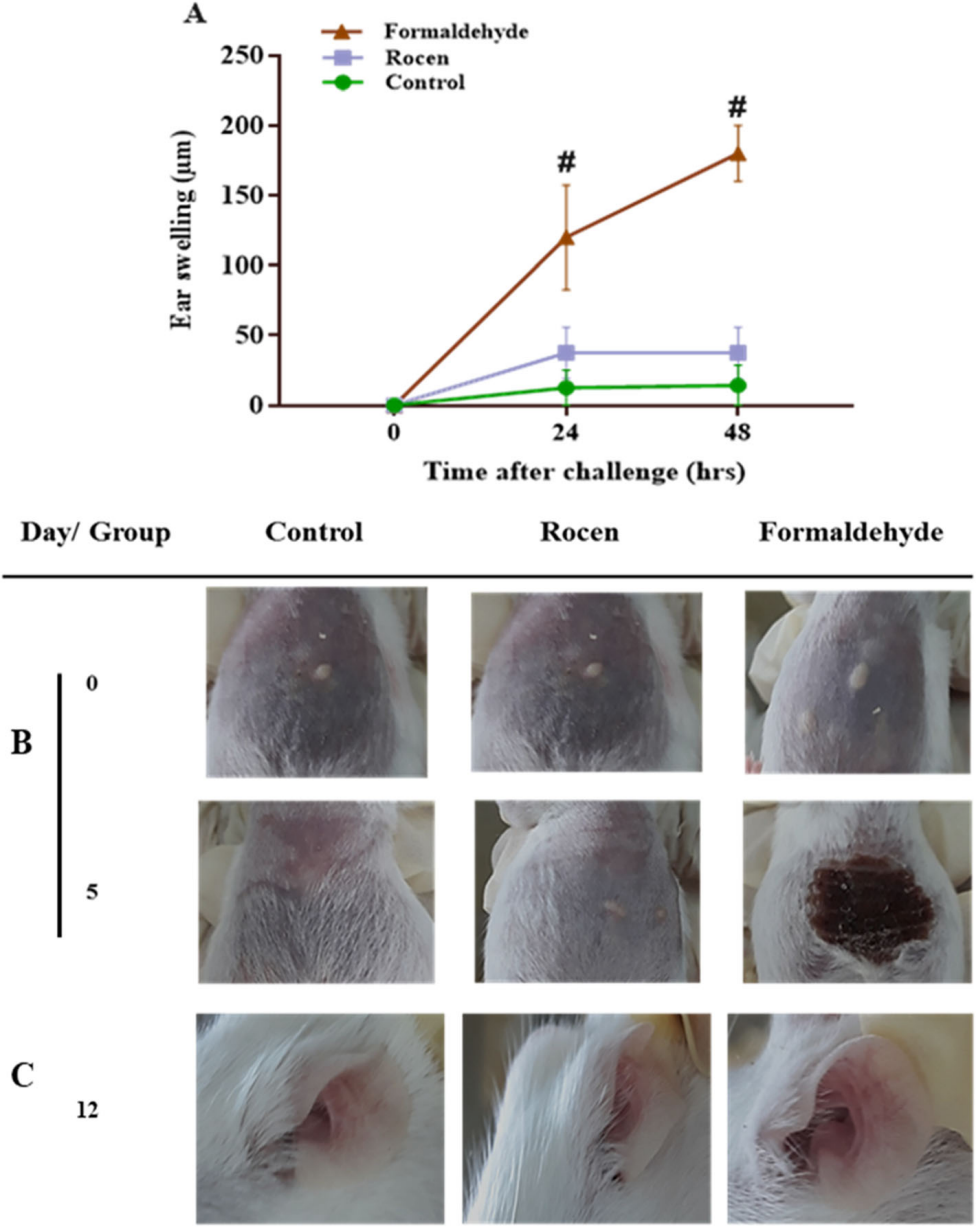
The demonstration of ROCEN allergenicity evaluation using MEST. **A** Swelling measurement of the left ear of the mice did not show any changes via ROCEN application. *n* = 10 for all groups (^**#**^ *P*< 0.0001, formaldehyde group compared to control group). **B** The induction stage through intradermal injections of the relative substances on the mice abdomen that were applied topically by rubbing gently. The lesion site was observable in the formaldehyde group on the 5th day of the experiment. **C** The representative mice ear shows a considerable ear redness in the formaldehyde group (sensitized mice) but without erythema or swelling in mice receiving ROCEN on the 12th day

The allergenicity potential of ROCEN was assessed using the MEST protocol (Fig. 3a, b, c). In the positive control group, topical application of formaldehyde caused a significant increase in ear thickness and redness of mice (*p*<0.0001) (Fig. 3a). ROCEN did not cause any sensitive reaction in the ear thickness and redness of mice (Fig. 3c).

### Skin severity reduction by ROCEN

**Fig. 4:**
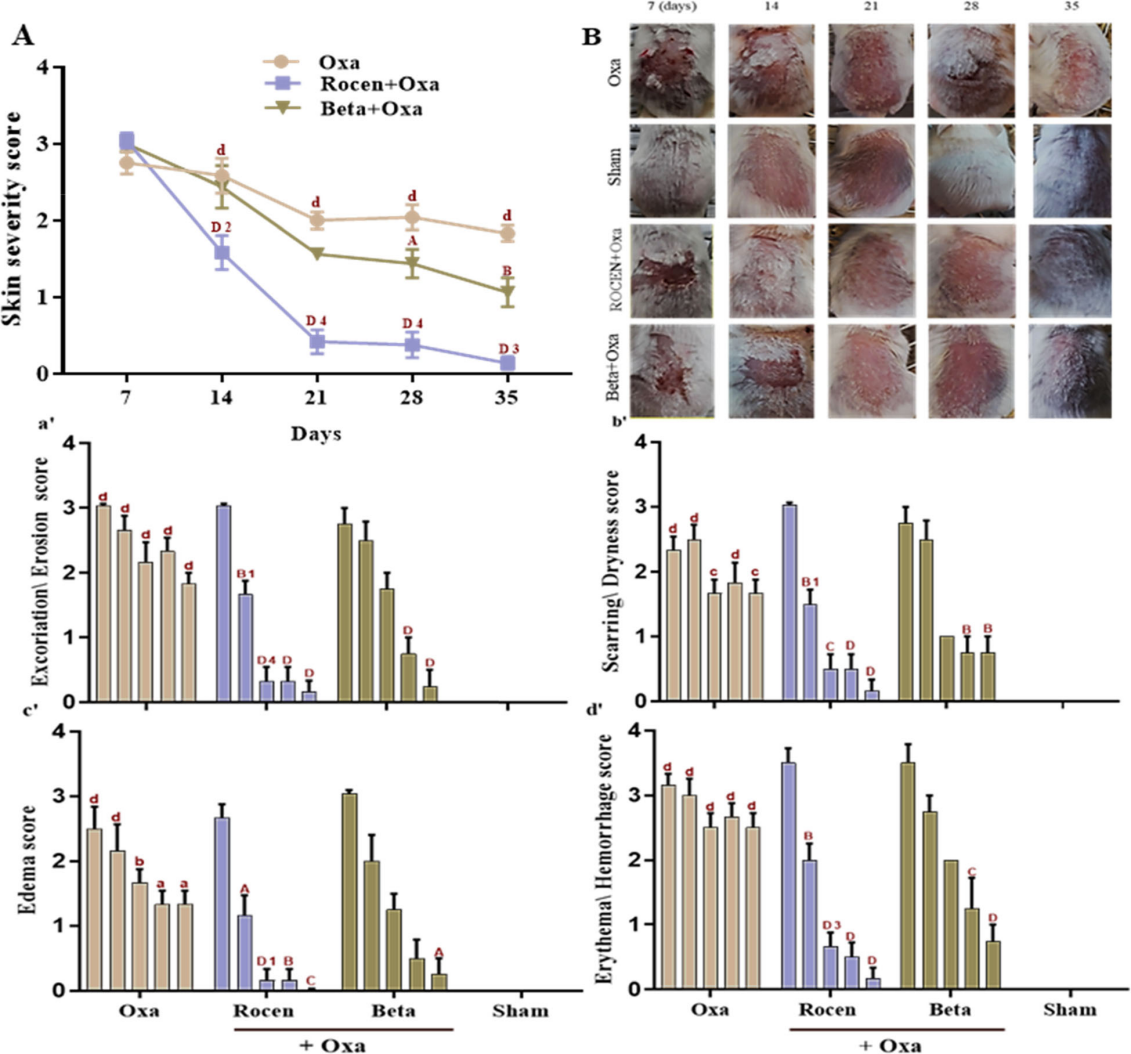
Indicative skin severity data of AD-like skin lesions and treatment effect by ROCEN in mice. **A** skin severity score of relative time points indicated a significant dermatitis healing by topical ROCEN rather than Beta in AD mice. The severity score was achieved by the sum of the relative dermatitis data (a’ (excoriation/erosion), b’ (scarring/dryness), c’ (edema), and d’ (erythema/ hemorrhage) scores) at 5 periods of experiment. **B**. Macroscopic features indicate that Oxa administration causes severe AD lesions at the different time intervals while ROCEN and Beta treatments recovered the lesion progress in mice. All data represented by mean ± SEM. **A**, **B**, **C**, **D**: treated groups compared with Oxa group. a, b, c, d: Oxa group compared with control group. 1, 2, 3, 4: ROCEN group compared with Beta group. Aa1; *p*<0.05, Bb2; *p*<0.01, Cc3; *p*<0.001, Dd4; *p*<0.0001

After repeated use of Oxa, skin dryness occurred with mild erythema and edema in AD mice followed by thick scars. These symptoms were not detectable in the sham group (Fig. 4a and b). As shown in Fig. 4Aa′b′c′d′, the dermatitis scores of the Oxa group were significantly higher compared to the control group at each time of the experiment (*p*<0.0001). In contrast, topical ROCEN significantly improved the dermatitis lesions and symptoms, even better than Beta (*p*<0.05). In addition, according to Fig. 4a, application of Beta as a standard drug caused a significant improvement in dermatitis only on the 28th (*p*<0.05) and 35th (*p*<0.01) days compared to the Oxa group. The details of the data scores related to the experiments are also shown in Fig. 4.

### Itching control by ROCEN

The behavior of the animals was analyzed on days 7, 14, 28, and 35 of the experimentation. The Oxa group showed a significant increase in scratching, grooming, and licking often 30 min after the Oxa application compared to the sham group (*p*<0.05) (Table 1). The scores of scratching and wiping were significantly lower in the ROCEN group versus the untreated Oxa group at all measurement times (*p*<0.05). Moreover, the results showed that ROCEN significantly improved the symptoms of eczema, even better than Beta (Table 1). Other findings are presented in Table 1.

**Table 1:** Scratching/wiping frequency data in treated AD mice. Data defined are as the mean ± SEM; *n* = 6 for all groups

Behavior type	Date	Oxa	Sham	Rocen + Oxa	Beta + Oxa
**No. of scratching** **(per 20 min)**	7th day	42.8 ± 6.8^**C**^	9.6 ± 3.8	14 ± 4.3^**b**^	18 ± 0.3^**a**^
	14th day	18.2 ± 4.1^**B**^	9 ± 4.5	10.5 ± 3.5^**a**^	13 ± 3.2
	28th day	108 ± 16.1^**D**^	7.5 ± 1	29.6 ± 6.1^**d**^	54.25 ± 18.5^**b**^
	35th day	113.6 ± 14^**D**^	10 ± 3	32.6 ± 10.3^**d**^	50.7 ± 15.3^**c**^
**No. of grooming** **(per 20 min)**	7th day	29.8 ± 8.8^**A**^	9.6 ± 3.8	8.2 ± 3.9^**b1**^	16.3 ± 2.1^**a**^
	14th day	21.2 ± 1.9^**A**^	14 ± 1.8	18.4 ± 2.7	18 ± 1.4
	28th day	67.4 ± 6.5^**D**^	6 ± 7	10.5 ± 1.9^**d1**^	30.5 ± 2.7^**d**^
	35th day	78 ± 12.5^**D**^	12.5 ± 2.5	53.6 ± 10.3^**a**^	39.7 ± 8.2^**b**^
**Time of licking (s)** **(per 20 min)**	7th day	341.8 ± 7^**D**^	74 ± 12.1	115 ± 41.3^**d2**^	229.4 ± 71.3
	14th day	174 ± 29.5	107 ± 48.6	129.6 ± 25.1	214.3 ± 131
	28th day	294.6 ± 39.2^**D**^	34.3 ± 4.5	40.3 ± 1.9^**d3**^	234.2 ± 19.5^**a**^
	35th day	340 ± 40.4^**D**^	12.5 ± 2.5	58.1 ± 22.3^**c2**^	229.5 ± 8.5

A,B,C,D: Oxa compared with control group. a,b,c,d: treated groups compared with Oxa group. 1,2,3,4: ROCEN compared with Beta group. Aa1; *p*<0.05, Bb2; *p*<0.01, Cc3; *p*<0.001, Dd4; *p*<0.0001

### Histological and immunohistochemical analysis

**Fig. 5:**
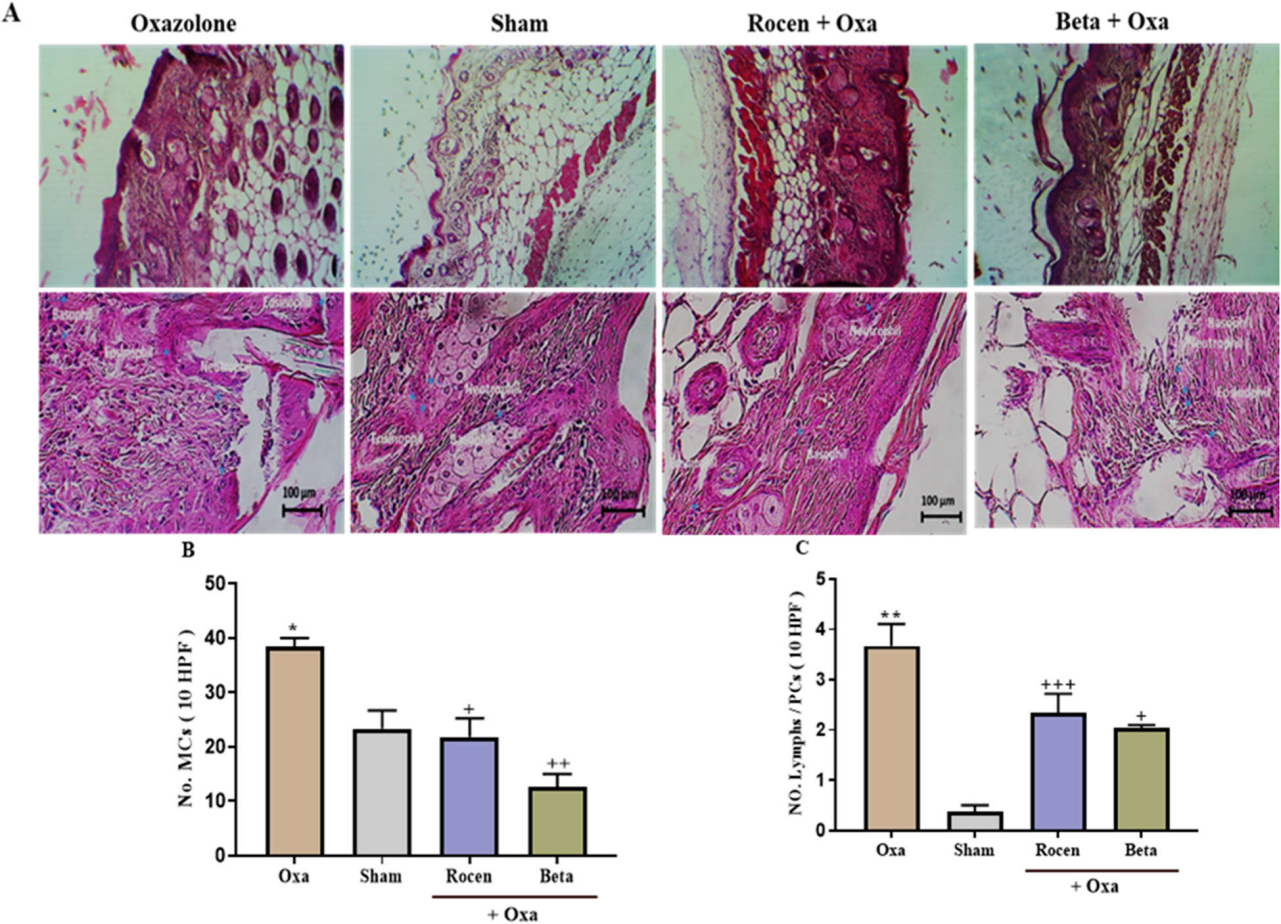
**A** Histopathological sections of Oxa-induced dermatitis and treated groups. The inflammatory cells were assessed under a light microscope. **B**. The number of MCs (**B**) and Lymphs/ plasma cells (**C**) were defined as the mean ± SEM; *n* = 6 for all groups. * When Oxa compared with sham group, and + treated group compared to Oxa group. *& + *p*< 0.05, **&++ *p*< 0.01, +++ *p*< 0.001

Histological changes of the dorsal skin of the mice were evaluated on the 36th day of the experiment. The lesion skin showed a significant (*p*<0.05) epithelial thickness, collagen deposition, angiogenesis, and leukocyte infiltration in the Oxa group (Table 2 & Fig. 5a). ROCEN significantly improved the histological parameters via increasing epithelial thickness and reducing collagen deposition and angiogenesis compared to the untreated Oxa group (*p*<0.05). Furthermore, the infiltration of inflammatory cells such as MCs (Fig. 5b) and Lymphs/PCs (Fig. 5c) was reduced significantly (*p*<0.05) by topical ROCEN, indicating more inflammation improvement compared to the Beta group.

**Table 2:** The histological parameters of mice skin subjected to AD in different experiments. Data showed are as mean ± SEM

Experiment	Epithelial thickness (μm)	Collagen deposition %	Angiogenesis No./ mm^**2**^	Leucocyte infiltration No./mm^**2**^
**Oxa**	67.74 ± 7.68^C^	52.49 ± 4.4^B^	4.8 ± 0.54^A^	1250 ± 85.63^C^
**Sham**	159.5 ± 33.84	37.01 ± 3.98	3.63 ± 0.66	913.33 ± 76.88
**ROCEN+ Oxa**	139.8 ± 12.42^b^	34.11 ± 3.74^b^	3.8 ± 0.23^a^	806 ± 51.92^d2^
**Beta + Oxa**	107.77 ± 9.77	43.9 ± 4.14	6.05 ± 1.1	1045 ± 51.85^a^

A,B,C,D: Oxa compared with control group. a,b,c,d: treated groups compared with Oxa group. 1,2,3,4: ROCEN compared with Beta group. Aa1; *p*<0.05, Bb2; *p*<0.01, Cc3; *p*<0.001, Dd4; *p*<0.0001

**Fig. 6:**
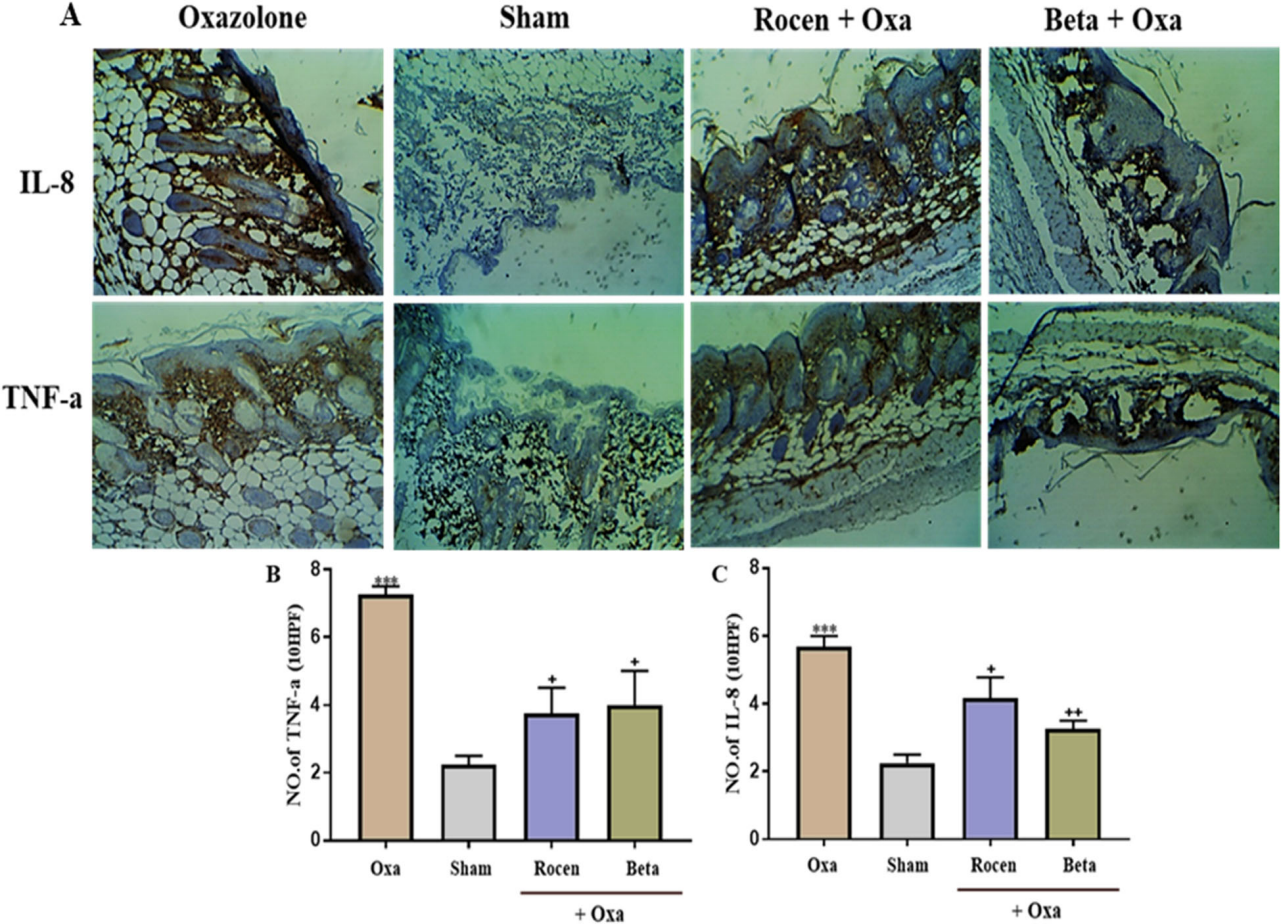
Data immunohistochemistry of cytokines related to topical treatment of AD mice by the drugs. **A** Representative sections of IHC evaluation related to IL-8 and TNF-α cytokines on the dorsal skin of AD mice. Photographs of TNF-α (**B**) and IL-8 (**C**) IHC analysis were defined as mean ± SEM. * Oxa compared with control group, + treated group compared with Oxa group. + *p*< 0.05, ++*p*< 0.01, ***p < 0.001

In addition, immunohistochemical staining (Fig. 6a) revealed a significant overproduction of TNF-α (Fig. 6b) and IL-8 (Fig. 6c) in the sensitized dermis after topical application of Oxa compared to the control (*p*< 0.001). Similar to Beta, topical ROCEN application significantly decreased TNF-α and IL-8 staining compared to the Oxa group (*p*<0.05), as shown in Fig. 6.

## Discussion

AD or eczema as a common chronic inflammatory skin disorder is associated with chronic pruritus, inflammation, and cell proliferation [1]. AD patients with systemic oral corticosteroid or even topical corticosteroid treatment like Beta may be potentially at risk of side effects of the drug [13, 29]. In this study, we compared the effect of topical Beta with ROCEN, a new anti-AD formulation, on a murine model.

ROCEN is prepared from natural oil [14] and lecithin in the liposomal structure and is well-known as a drug supplementation without any adverse effects in pharmacy [16]. ROCEN not only has anti-inflammatory and analgesic effects in experimental reports but also may play role in barrier homeostasis of the skin due to its permeability property [16, 19]. The safety of topical ROCEN hydrogel is an important factor for its long-term application in allergic dermatitis. However, pharmaceutical criteria recommend more clinic studies on possible sensitization by this drug.

In the current study, treatment with topical ROCEN was associated with an improvement in all important dermatitis symptoms and ameliorated itching and wiping behaviors in mice subjected to eczema. Itching is the major symptom of inflammatory AD that impairs life quality in the patients. Itching stimulates the scratch reflex and increases the desire to scratch, resulting in exacerbation of the skin lesions, secondary infections, and sleep disturbances [30, 31]. Therefore, attenuation of itching is believed to be one of the fundamental approaches in controlling skin lesion progression in an eczematous state [32].

Histopathologically, eczematous lesions are characterized by epidermal hyperplasia and dermal inflammatory cell infiltration with increased numbers of monocytes, macrophages, mast cells, basophils, and eosinophils [33, 34] as well as neutrophils in the early inflammatory reaction [35]. Angiogenesis occurs physiologically in the wound healing process and also during inflammatory diseases [36]. In addition, mast cells may stimulate angiogenesis via the release of proangiogenic factors in AD lesions [37]. Besides, fibrocystic accumulation in skin fibrogenesis may lead to the production of cytokines that induce collagen deposition in the lesion site [38–40]. It has been shown that suppression of fibrogenesis reduces proangiogenic mediators, resulting in decreased angiogenesis and activation of T cells [41].

In this study, daily application of ROCEN significantly modulated angiogenesis and collagen deposition and reduced the infiltration of leucocytes such as eosinophils in the skin tissue of AD mice compared to Beta.

In the present study, TNF-α [42] and IL-8 [1, 43], as proinflammatory cytokines involved in allergic contact dermatitis, were evaluated histochemically after 36 days treatment with ROCEN. It has been proposed that IL-8 is a non-specific mediator with mitogenic activity in keratinocytes that induces inflammation or immunogenic factors after stimulation with contact sensitizers and irritants [43]. Moreover, the upregulation of TNF-α along with other inflammatory cytokines contributes to epidermal abnormality and skin barrier dysfunctionality, resulting in thickened skin and scale formation [1, 10]. Danso et al. proposed that barrier disruption in AD increased skin susceptibility to penetration of allergens through the skin layers, initiating a Th2 immune response and TNF-α in the epidermis [10].

Our findings suggest a hypothesis that topical ROCEN can potentially reduce pro-inflammatory cytokines such as TNF-α and IL-8, resulting in lipid homeostasis of the skin barrier in the lesion site and improvement of dermatitis symptoms. However, further evaluation of other hallmarks or mechanisms involved in AD disease will be valuable to confirm anti-allergic properties of ROCEN.

## Conclusion

The findings showed the therapeutic benefits of topical ROCEN in controlling AD, which was even better than Beta. ROCEN reduced tissue damage and dermatitis symptoms like pruritus, and downregulated inflammatory cytokines associated with a contact stimulus. Topical ROCEN may be applicable in the clinical setting instead of current AD drugs to minimize drug side effects.

## Data Availability

The datasets analyzed during the current study are available from the corresponding author on reasonable request.

## References

[1] Lee GR, Maarouf M, Hendricks AK, Lee DE, Shi VY (2019). Current and emerging therapies for hand eczema. Dermatol Ther.

[2] Coenraads PJ (2012). Hand eczema. N Engl J Med.

[3] Pastore S, Mascia F, Giustizieri ML, Giannetti A, Girolomoni G (2000). Pathogenetic mechanisms of atopic dermatitis. Arch Immunol Ther Exp.

[4] Leung DY (2000). Atopic dermatitis: new insights and opportunities for therapeutic intervention. J Allergy Clin Immunol.

[5] Akdis CA, Akdis M, Trautmann A, Blaser K (2000). Immune regulation in atopic dermatitis. Curr Opin Immunol.

[6] Čelakovská J, Bukač J, Vaňková R, Cermakova E, Krcmova I, Krejsek J, Andrýs C (2020). Cluster analysis of molecular components in 100 patients suffering from atopic dermatitis according to the ISAC multiplex testing. Food Agric Immunol.

[7] Kang JS, Lee K, Han S-B, Ahn J-M, Lee H, Han MH, Yoon YD, Yoon WK, Park S-K, Kim HM (2006). Induction of atopic eczema/dermatitis syndrome-like skin lesions by repeated topical application of a crude extract of Dermatophagoides pteronyssinus in NC/Nga mice. Int Immunopharmacol.

[8] Peiser M, Tralau T, Heidler J, Api AM, Arts JH, Basketter DA, English J, Diepgen TL, Fuhlbrigge RC, Gaspari AA (2012). Allergic contact dermatitis: epidemiology, molecular mechanisms, in vitro methods and regulatory aspects. Current knowledge assembled at an international workshop at BfR, Germany. Cell Mol Life Sci.

[9] Sticherling M, Bornscheuer E, Schroder JM, Christophers E (1992). Immunohistochemical studies on NAP-1/IL-8 in contact eczema and atopic dermatitis. Arch Dermatol Res.

[10] Danso MO, Van Drongelen V, Mulder A, Van Esch J, Scott H, Van Smeden J, El Ghalbzouri A, Bouwstra JA (2014). TNF-α and Th2 cytokines induce atopic dermatitis–like features on epidermal differentiation proteins and stratum corneum lipids in human skin equivalents. J Investig Dermatol.

[11] Watanabe N, Hanabuchi S, Soumelis V, Yuan W, Ho S, de Waal MR, Liu Y-J (2004). Human thymic stromal lymphopoietin promotes dendritic cell–mediated CD4+ T cell homeostatic expansion. Nat Immunol.

[12] Berke R, Singh A, Guralnick M (2012). Atopic dermatitis: an overview. Am Fam Physician.

[13] Hengge UR, Ruzicka T, Schwartz RA, Cork MJ (2006). Adverse effects of topical glucocorticosteroids. J Am Acad Dermatol.

[14] Henrotin YE, Deberg MA, Crielaard J-M, Piccardi N, Msika P, Sanchez C (2006). Avocado/soybean unsaponifiables prevent the inhibitory effect of osteoarthritic subchondral osteoblasts on aggrecan and type II collagen synthesis by chondrocytes. J Rheumatol.

[15] Au R, Al-Talib T, Au A, Phan P, Frondoza C (2007). Avocado soybean unsaponifiables (ASU) suppress TNF-α, IL-1β, COX-2, iNOS gene expression, and prostaglandin E2 and nitric oxide production in articular chondrocytes and monocyte/macrophages. Osteoarthr Cartil.

[16] Goudarzi R, Nasab ME, Saffari PM, Zamanian G, Park CD, Partoazar A. Evaluation of ROCEN on burn wound healing and thermal pain: transforming growth factor-beta1 activation. Int J Low Extrem Wounds. 2020. 10.1177/1534734620915327. 10.1177/1534734620915327 32308073

[17] Kianvash N, Bahador A, Pourhajibagher M, Ghafari H, Nikoui V, Rezayat SM, Dehpour AR, Partoazar A (2017). Evaluation of propylene glycol nanoliposomes containing curcumin on burn wound model in rat: biocompatibility, wound healing, and anti-bacterial effects. Drug Deliv Transl Res.

[18] Partoazar A, Kianvash N, Darvishi MH, Nasoohi S, Rezayat SM, Bahador A (2016). Ethosomal curcumin promoted wound healing and reduced bacterial Flora in second degree burn in rat. Drug Res.

[19] Goudarzi R, Amini S, Dehpour AR, Partoazar A (2019). Estimation of anti-inflammatory and analgesic effects of topical NANOCEN (Nanoliposomal Arthrocen) on mice. AAPS PharmSciTech.

[20] Nasab ME, Takzaree N, Saffari PM, Partoazar A (2019). In vitro antioxidant activity and in vivo wound-healing effect of lecithin liposomes: a comparative study. J Comp Eff Res.

[21] Saffari PM, Alijanpour S, Takzaree N, Sahebgharani M, Etemad-Moghadam S, Noorbakhsh F, Partoazar A (2020). Metformin loaded phosphatidylserine nanoliposomes improve memory deficit and reduce neuroinflammation in streptozotocin-induced Alzheimer's disease model. Life Sci.

[22] Zamanian G, Partoazar A, Tavangar SM, Rashidian A, Mirzaei P, Niaz Q, Sharifi K, Dehpour AR, Jazaeri F (2020). Effect of phosphatidylserine on cirrhosis-induced hepatic encephalopathy: response to acute endotoxemia in cirrhotic rats. Life Sci.

[23] Thorne PS, Hawk C, Kaliszewski SD, Guiney PD (1991). The noninvasive mouse ear swelling assay: I. refinements for detecting weak contact sensitizers. Fundam Appl Toxicol.

[24] Goindi S, Kumar G, Kaur A (2014). Novel flexible vesicles based topical formulation of levocetirizine: in vivo evaluation using oxazolone-induced atopic dermatitis in murine model. J Liposome Res.

[25] Matsuda H, Watanabe N, Geba GP, Sperl J, Tsudzuki M, Hiroi J, Matsumoto M, Ushio H, Saito S, Askenase PW (1997). Development of atopic dermatitis-like skin lesion with IgE hyperproduction in NC/Nga mice. Int Immunol.

[26] Shimada SG, LaMotte RH (2008). Behavioral differentiation between itch and pain in mouse. Pain.

[27] Sheu MY, Fowler AJ, Kao J, Schmuth M, Fluhr JW, Man M-Q, Elias PM, Feingold KR, Schoonjans K, Auwerx J (2002). Topical peroxisome proliferator activated receptor-α activators reduce inflammation in irritant and allergic contact dermatitis models. J Investig Dermatol.

[28] Partoazar A, Seyyedian Z, Zamanian G, Saffari PM, Muhammadnejad A, Dehpour AR, Goudarzi R (2021). Neuroprotective phosphatidylserine liposomes alleviate depressive-like behavior related to stroke through neuroinflammation attenuation in the mouse hippocampus. Psychopharmacology.

[29] Diepgen TL, Agner T, Aberer W, Berth-Jones J, Cambazard F, Elsner P, McFadden J, Coenraads PJ (2007). Management of chronic hand eczema. Contact Dermatitis.

[30] Koblenzer CS (1999). Itching and the atopic skin. J Allergy Clin Immunol.

[31] Bender BG, Leung SB, Leung DY (2003). Actigraphy assessment of sleep disturbance in patients with atopic dermatitis: an objective life quality measure. J Allergy Clin Immunol.

[32] Wahlgren C-F (1991). Itch and atopic dermatitis: clinical and experimental studies. Acta Derm Venereol Suppl.

[33] Straumann A, Simon HU (2004). The physiological and pathophysiological roles of eosinophils in the gastrointestinal tract. Allergy.

[34] Mihm MC, Soter NA, Dvorak HF, Austen KF (1976). The structure of normal skin and the morphology of atopic eczema. J Invest Dermatol.

[35] Ott NL, Gleich GJ, Peterson EA, Fujisawa T, Sur S, Leiferman KM (1994). Assessment of eosinophil and neutrophil participation in atopic dermatitis: comparison with the IgE-mediated late-phase reaction. J Allergy Clin Immunol.

[36] Ferrara N. Vascular endothelial growth factor: pathophysiology and clinical implications. In: Angiogenesis. Boca Raton: CRC Press; 2006. p. 15–50.

[37] Detoraki A, Granata F, Staibano S, Rossi F, Marone G, Genovese A (2010). Angiogenesis and lymphangiogenesis in bronchial asthma. Allergy.

[38] Quan TE, Cowper S, Wu S-P, Bockenstedt LK, Bucala R (2004). Circulating fibrocytes: collagen-secreting cells of the peripheral blood. Int J Biochem Cell Biol.

[39] Schmidt M, Sun G, Stacey MA, Mori L, Mattoli S (2003). Identification of circulating fibrocytes as precursors of bronchial myofibroblasts in asthma. J Immunol.

[40] Metz C (2003). Fibrocytes: a unique cell population implicated in wound healing. Cell Mol Life Sci.

[41] Chesney J, Bacher M, Bender A, Bucala R (1997). The peripheral blood fibrocyte is a potent antigen-presenting cell capable of priming naive T cells in situ. Proc Natl Acad Sci.

[42] Kiehl P, Falkenberg K, Vogelbruch M, Kapp A (2001). Tissue eosinophilia in acute and chronic atopic dermatitis: a morphometric approach using quantitative image analysis of immunostaining. Br J Dermatol.

[43] Mohamadzadeh M, Miiller M, Hultsch T, Enk A, Saloga J, Knop J (1994). Enhanced expression of IL-8 in normal human keratinocytes and human keratinocyte cell line HaCaT in vitro after stimulation with contact sensitizers., tolerogens and irritants. Exp Dermatol.

